# Using Sibling Designs to Understand Neurodevelopmental Disorders: From Genes and Environments to Prevention Programming

**DOI:** 10.1155/2015/672784

**Published:** 2015-07-15

**Authors:** Mark Wade, Heather Prime, Sheri Madigan

**Affiliations:** ^1^Department of Applied Psychology and Human Development, University of Toronto, 252 Bloor Street W., Toronto, ON, Canada M5S 1V6; ^2^Department of Psychology, University of Calgary, 2500 University Drive NW, Calgary, AB, Canada T2N 1N4

## Abstract

Neurodevelopmental disorders represent a broad class of childhood neurological conditions that have a significant bearing on the wellbeing of children, families, and communities. In this review, we draw on evidence from two common and widely studied neurodevelopmental disorders—autism spectrum disorder (ASD) and attention-deficit hyperactivity disorder (ADHD)—to demonstrate the utility of genetically informed sibling designs in uncovering the nature and pathogenesis of these conditions. Specifically, we examine how twin, recurrence risk, and infant prospective tracking studies have contributed to our understanding of genetic and environmental liabilities towards neurodevelopmental morbidity through their impact on neurocognitive processes and structural/functional neuroanatomy. It is suggested that the siblings of children with ASD and ADHD are at risk not only of clinically elevated problems in these areas, but also of subthreshold symptoms and/or subtle impairments in various neurocognitive skills and other domains of psychosocial health. Finally, we close with a discussion on the practical relevance of sibling designs and how these might be used in the service of early screening, prevention, and intervention efforts that aim to alleviate the negative downstream consequences associated with disorders of neurodevelopment.

## 1. Neurodevelopmental Disorders: Overview and Frame

Neurodevelopmental disorders are brain-based disorders of growth and development that have a significant impact on children's mental, emotional, and psychosocial health. Among the most common and widely studied neurodevelopmental disorders are autism spectrum disorder (ASD) and attention-deficit hyperactivity disorder (ADHD [1, 2]). The etiology of these conditions has yet to be completely mapped; however, there is strong evidence for a role of both genetic and environmental influences operating together in their pathogenesis [3–7]. As human brain development is shaped by continuous interactions between genetic and environmental factors, neurocognitive functioning may be considered an intermediate phenotype linking genes (and environments) to symptom manifestations of disorders [8]. A major problem faced by developmental scientists and clinicians is that, after symptoms have emerged and a diagnosis has been made, it is difficult to disentangle the relative (and joint) contribution of these factors. The fallout is a propensity to intervene with remedial programs that target symptoms rather than the factors and processes that predispose to disordered phenotypes. Although this is a reasonable and necessary endeavour, there are significant social and economic costs associated with the management of these conditions, related to lost productivity, medical care, and special education services [9–14]. In addition, children with either ASD or ADHD experience more social, familial, academic, and occupational difficulties [15, 16], suggesting tremendous personal burden as a result of persistent problems across the lifespan.

Given the individual, familial, and societal burden associated with neurodevelopmental disorders, there is a crucial need for methods of early detection to enable prevention programming prior to the emergence of fully fledged symptoms [17, 18]. The goal of this review is to discuss how genetically sensitive sibling designs offer one such method for understanding the basis and risk of ASD and ADHD, as well as their overlap, and to highlight the utility of these designs in crafting prevention and intervention programs that may help ameliorate the negative downstream sequelae of neurodevelopmental morbidity. Here, we operationalize* sibling design* as any genetically informed design that capitalizes on a comparison or evaluation of traits in genetically similar individuals, including monozygotic or dizygotic twins, nontwin full siblings, or half-siblings.

## 2. Autism Spectrum Disorder (ASD)

### 2.1. Overview of ASD

The prevalence of ASD is estimated at around 1% of the population [19–21], with representation across all racial, ethnic, and socioeconomic groups. Notably, the prevalence of ASD has increased over the last 15 years [20, 22]. This increase has almost certainly been shaped by a broadening of diagnostic criteria as well as increased awareness and improved early detection of ASD [23]. Whether there is also a genuine secular increase in ASD is currently not clear from the extant literature [24]. The previous version of the Diagnostic and Statistical Manual (DSM-IV) characterized autism by a triad of problems, including impaired social interaction, social communication, and restricted/repetitive patterns of behaviour, interest, or activities. The new version of this manual (DSM-5) only demarcates two clusters, with social communication and interaction under one umbrella and repetitive behaviours under the other. Also, DSM-5 introduced Social Communication Disorder, for which there is some concern that individuals previously meeting criteria for Asperger's Disorder or Pervasive Developmental Disorder-Not Otherwise Specified (PDD-NOS) could be inadvertently classified [25]. Although this review will not elaborate on this point further, an important caveat is that most studies reported herein were conducted using DSM-IV guidelines. Where possible, we incorporate the most up-to-date literature using DSM-5 criteria.

### 2.2. Sibling Studies of ASD

Over the last two decades, there has been a shift towards viewing ASD symptomatology along a quantitative spectrum as opposed to representing a discrete disorder [26]. In this regard, diagnosis is ultimately the categorical distinction of highly variable quantitative cognitive and behavioural traits. This relatively new conceptualization of autism as a spectrum disorder has raised questions about its pathogenesis, which in turn has demanded the application of a variety of methodological approaches for examining its etiology. One such methodology is the sibling design, which explores the siblings of children with an ASD diagnosis in order to understand the genetic and environmental contributors to the disorder, as well as to delineate other risk and protective factors and early markers associated with ASD in high-risk children. Ultimately, the information obtained from such designs positions researchers, clinicians, and policymakers to create and implement prevention efforts that target children who are susceptible to the disorder.

#### 2.2.1. Twin Designs of ASD

The genetic pathophysiology of ASD is supported by three primary sibling-related designs. The first, called a* twin study*, compares monozygotic (MZ) twins (who share 100% of their genetic information) to dizygotic (DZ) twins (who like nontwin siblings share 50% of their genes but like MZ twins share the same intrauterine environment). In a twin study, if the concordance rate among MZ twins is higher than that among DZ twins, then genetic influences are assumed. This is because the primary differences between MZ and DZ twins, aside from nonshared factors (e.g., experiences outside the home, friends, etc.), are genetically mediated. Twin studies to date yield heritability estimates of ASD between 70 and 80% [27–29]. Aside from one recent study suggesting a significant contribution of shared environmental factors to ASD (experiences that are common to both twins, e.g., socioeconomic status and parental mental health problems [4]), these studies have generally reported small or nonsignificant shared environmental effects. In general, these studies of twins are indicative of strong familial, or genetic, risk of developing ASD [30]. Also, twin studies have shown that ASD traits are moderately to highly heritable whether measured continuously or at extreme, clinical levels, with the overarching suggestion that ASD may best be considered along a continuum that varies genetically throughout the population [26, 29].

The presumed effect of genetic influences on development and behaviour is through endophenotypic variability in brain functioning and neurocognition [31]. In terms of neuroanatomy, clinically concordant MZ twins show more similarity in structural neuroanatomy than discordant MZ twins, despite the fact that discordant twins are more similar than nontwins in various brain regions [32, 33]. This finding cannot be easily explained by differences in heritability factors, as all MZ twins share 100% of their genes. However, it is interesting to note that approximately 65–75% of MZ twins are monochorionic (MC)—meaning they share the same placenta—while the remaining 25–35% of MZ twins (and 100% of DZ twins) are dichorionic (DC), having different placentas. We know of no twin studies that have differentiated between MC-MZ and DC-MZ twins, although mathematical equations have been derived to predict this variability [34]. These mathematical models suggest that MC-MZ twins, who have virtually identical intrauterine environments in addition to genetic likeness, would have concordance rates at about 95–100%, while DC-MZ twins would exhibit concordance rates comparable to DZ twins or ordinary nontwin siblings. If true, this suggests that an intrauterine trigger or insult that is more similarly shared by MC-MZ twins may generate more comparable deficits in neural functioning that underlie the social and cognitive impairments that characterize ASD. Alternatively, DC-MZ twins, who are more likely to be discordant for a diagnosis, may evince comparable neuroanatomy in some areas but not others, which explain the relatively lower symptom overlap, perhaps at subclinical levels that do not warrant full diagnosis. These purported models require explicit empirical investigation in future studies.

In sum, these studies point to a strong genetic pathophysiology of ASD, and studies that link the genes involved in ASD to discrete brain circuits that support cognitive functions will prove critical in advancing our knowledge of these complex and as-yet unidentified mechanisms of development [35].

#### 2.2.2. Sibling Recurrence Rates of ASD

The second sibling approach to studying the etiology of ASD is to examine* recurrence rates* in nontwin sibling pairs. These methods seek to determine the likelihood of an ASD diagnosis in the younger siblings of a child with an existing ASD diagnosis. Recent studies report that when followed from infancy to early childhood, the risk of developing ASD is between 10 and 20% for siblings of children with ASD compared to about 1% for siblings of a typically developing child [36–38]. Moreover, two studies of over 5,000 nontwin sibling pairs showed that the recurrence rate of ASD in full siblings (~10%) is about twice that of half-siblings (~5%), providing evidence for an apparent dose-response relationship between degree of genetic relatedness and risk of ASD in nontwin siblings [39, 40].

Interestingly, findings from twin studies suggest that the recurrence risk in DZ twins is about 30% [27], considerably higher than that observed in nontwin siblings. Given that a shared* in utero* environment is one of the foremost factors differentiating DZ from nontwin siblings, these findings again suggest that intrauterine factors, such as the elicitation of deleterious antibodies or differential recruitment of maternal resources, may represent additional sources of variability contributing to the risk of ASD. Indeed, a number of prenatal environmental agents have been linked to an increased risk of ASD, including maternal medications during pregnancy (e.g., thalidomide and valproic acid [41, 42]), maternal viral infections (e.g., cytomegalovirus [43]), and possibly prenatal exposure to organophosphates [44] and phthalates [45]. To the extent that the intrauterine environment is in fact more similar for DZ twins than for nontwin siblings, it is also possible that the increased recurrence risk of DZ twins compared to nontwin siblings reflects a shared genetic risk interacting with shared prenatal exposures [46, 47]. In sum, not only is the recurrence risk of ASD higher in siblings than nonsiblings, but also there appears to be an increasingly heightened risk as a function of genetic relatedness and the extent of environmental similarity that may manifest in distinct neural, cognitive, and socioemotional profiles that typify ASD [48].

#### 2.2.3. Prospective Tracking of Infants at High Risk of ASD

The third stream of research utilizing sibling designs are those that* prospectively track* infants at risk for ASD, which is usually operationally defined as having an older sibling with ASD. By prospectively following high-risk infants, researchers can compare those that do and do not develop ASD to one another as well as to a control group (i.e., infants with typically developing siblings). They can also compare siblings of children with ASD to the siblings of typically developing children on core neuropsychological, socioemotional, and behavioural traits to identify early markers of ASD. It is also possible to track the trajectories of children at risk of ASD to ascertain long-term neurodevelopmental outcomes and whether early behavioural markers are associated with later diagnostic classification.

Studies tracking siblings at risk of ASD have shown that the extent of their early difficulties is predictive of later social and behavioural problems and may also differentiate them from the siblings of typically developing children. For instance, in siblings of children with ASD, initial levels of joint attention (at 15 months) predicted the extent of social impairment and ASD diagnosis at 34 months [49] as well as language skills at age 5 [50]. In this same sample, 5-year-old children with an ASD sibling showed vulnerabilities on measures of executive functioning, social cognition, and repetitive behaviour, as compared to siblings of typically developing children [51]. No group differences were found in various other domains measured at age 5 (i.e., global IQ, language, and behavioural problems). These results suggest that the siblings of children with ASD may demonstrate comparatively more problems in discrete neurocognitive domains but not others, even in the absence of an ASD diagnosis. Other research groups have reported similar findings [49, 52, 53]. In general, the presence of ASD characteristics (albeit at a less severe level) in the siblings of children with ASD has led to the widely accepted notion of a “broad ASD phenotype” [50], which is also consistent with dimensional approaches to diagnosis. New lines of evidence are emerging which suggest that the nonaffected siblings of individuals with ASD demonstrate similar patterns of activation in the neural regions underlying key social-communicative impairments of ASD, such as gaze fixation [51] and face processing [54]. These results suggest that these (and other) phenotypes may represent functional trait markers of genetic susceptibility to ASD and may be expressed broadly in the relatives of individuals with ASD.

Perhaps the greatest benefit of prospective studies of siblings of children with ASD is to determine the temporal onset of cognitive/behavioural problems in children who themselves go on to develop ASD. These infant sibling studies have revealed reliable differences in language [55], orienting to name [56], nonverbal problem solving [57], joint attention [58, 59], imitation, social smiling, and social affect [60] in the second year of life. However, these and other studies [61] have generally failed to differentiate at-risk and non-at-risk children on the basis of cognitive and behavioural markers at 6 months or earlier, suggesting that social-cognitive abilities may be largely intact in the first year of life. Moreover, differences in social and communicative abilities may decline over the first 3 years of life in at-risk siblings who go on to develop ASD [62], suggesting a need to carefully track these siblings on a number of cognitive and behavioural markers early in development.

### 2.3. Summary and Future Directions for ASD Sibling Research

Studies employing sibling-based designs in the study of ASD have expanded our knowledge of the genetic epidemiology of the disorder and also help to clarify how genes and environments may operate in tandem in the pathogenesis of neurodevelopment. Twin designs suggest a high genetic contribution to ASD, a finding that is now well established. Further, research examining recurrence risk of ASD (either in twin or in nontwin siblings) suggests that not only is the degree of genetic relatedness associated with risk of ASD, but also environmental exposures may epigenetically regulate the expression of genes that modulate ASD symptomatology. In this regard, the joint circumstance of genetic vulnerability alongside exposure to teratogenic or neurotoxic agents may particularly predispose children to poor neurodevelopmental outcomes. Moreover, sibling studies that prospectively track infants have been useful in elucidating early markers of not only the risk of future ASD diagnosis, but also the risk of discrete neuropsychological skill deficits in the absence of ASD classification. The latter finding suggests that siblings of children with ASD may benefit from early prevention and monitoring.

While our knowledge of overall genetic and symptom risk related to ASD has been fostered by sibling studies, there remain gaps in our understanding of the discrete molecular genetic variants that create vulnerability to ASD among siblings, as well as how variability in neural functioning serves as an endophenotypic link between genetic risk and behavioural manifestations of the disorder. Future research in this area that compares MZ, DZ, nontwin full siblings, and half-siblings will shed light on whether the apparent gradient relationship between degree of genetic relatedness and risk of ASD also applies to cognitive processing and neural functioning that supports the behavioural and social-emotional faculties associated with ASD.

## 3. Attention-Deficit Hyperactivity Disorder (ADHD)

### 3.1. Overview of ADHD

ADHD is the most common childhood neurodevelopmental disorder, affecting approximately 5% of school-age children [63]. These prevalence estimates appear to have remained quite stable over time and are relatively ubiquitous across geographic location. According to the DSM-5, ADHD is characterized by a persistent pattern of inattention and/or hyperactivity/impulsivity. ADHD is associated with other cognitive impairments (i.e., executive functioning) [64] as well as a myriad of comorbid psychiatric problems across the lifespan [65–67], together causing significant impairment in academic, familial, and peer functioning. ADHD, like ASD, represents a major public health concern, with impairing symptoms persisting into adulthood in 65% of individuals [68] and the pooled prevalence in adulthood of about 2.5% [69]. Thus, early identification and intervention are essential to alleviate the long-term effects of ADHD and its associated neuropsychiatric morbidities.

### 3.2. Sibling Studies of ADHD

ADHD has an overlapping yet distinct neurocognitive profile to ASD [70]. Despite this, the application of sibling designs to the study of ADHD, especially those examining recurrence rates and prospective tracking of infants, has been explored far less than in ASD. Nonetheless, the existing evidence is generally consistent with studies on ASD, which is helpful in understanding both the unity and the diversity of neurodevelopmental disorders.

#### 3.2.1. Twin Designs of ADHD

Twin studies suggest that heritability of ADHD is high (*h* ^2^ = .75 to .91), whether defined according to strict clinical cut-offs or along a broad phenotypic continuum [71, 72], with limited shared environmental effects. These results, like those of ASD, suggest that genetic liability for ADHD traits exists across the entire population. Moreover, research suggests that executive functioning, one of the key impairments in ADHD, is almost entirely genetic in origin and that liability towards ADHD strongly involves EF deficits [73, 74]. In a sample of DZ twins discordant for ADHD diagnosis, it was shown that unaffected cotwins of children with ADHD were significantly impaired in many neuropsychological domains, including executive functioning, processing speed, and arousal regulation compared to controls, even after controlling for subclinical levels of ADHD symptoms [75]. These results suggest that neurocognitive deficits in these domains may serve as plausible endophenotypes in the etiology of ADHD. Although beyond the scope of this review, a number of molecular genetic studies have been conducted now which implicate many individual genes in the pathogenesis of ADHD [76, 77]. These studies suggest that the same risk alleles that contribute to behavioural variability in the general population also confer risk of diagnosis of ADHD [78, 79].

As with studies of ASD, sibling designs suggest that genetic liabilities towards neurodevelopmental morbidities are likely mediated by neurocognitive functioning. For example, a twin study by Anokhin et al. [80] demonstrated that 60% of the variance in midfrontal (anterior cingulate cortex) activity is genetically mediated, suggesting that there may be heritable individual differences in neural processing related to neurobehavioural functioning (also see [81]). Among MZ twins, those with high levels of ADHD traits show volume loss in orbitofrontal and inferior dorsolateral prefrontal cortex [82]. Decreased activation in left dorsolateral prefrontal cortex and right parietal lobe has also been observed during executive processing tasks [83]. Together, these results suggest that different brain regions within a distributed network are influenced by genetic factors and may be related to neurocognitive markers of ADHD. On the other hand, recent imaging genetic studies suggest that while certain behavioural markers of ADHD may be to some extent heritable, the heritability of the functional neural networks that underlie these cognitive abilities is less certain [84]. More studies are needed to flesh out the meditational role of structural/functional neuroanatomy in the link between genetic variability and discrete cognitive impairments that underlie neurodevelopmental disorders.

#### 3.2.2. Sibling Recurrence Rates of ADHD

The recurrence rate in nontwin siblings of children with ADHD has been shown to be about 13% [85] and as high as 30% [86], higher than population prevalence rates of 5% [87]. Indeed, ADHD status is associated with sibling ADHD traits, whether measured narrowly or as a dimensional construct [85], suggesting that the siblings of children with ADHD may be at risk of developing not only ADHD, but also subclinical symptoms of the disorder [85]. Such genetic risk makes the study of siblings of affected children a viable channel for understanding pathways of neurodevelopment.

#### 3.2.3. Prospective Tracking of Infants at High Risk of ADHD

As with ASD, studies suggest that the siblings of children with ADHD are at a relatively higher risk of developing ADHD or related symptoms compared to those whose siblings do not have ADHD. For instance, Nigg et al. [88] showed that the parents and siblings of children with ADHD (especially combined type) were more likely to have an ADHD diagnosis compared to controls and had more neuropsychological impairments, even after controlling for parent/sibling ADHD status. On the other hand, prospective studies of at-risk children are relatively scarce compared to studies on ASD. In one notable study by Faraone and colleagues [89], siblings of children with ADHD showed significantly higher rates of disruptive behaviour, anxiety, depression, and school-related difficulties compared to the siblings of non-ADHD children. These results corroborate early reports of the family-genetic risk of ADHD and intellectual impairment in the siblings of children with ADHD [90, 91]. They are also consistent with related avenues of research showing that the siblings of children with ADHD are at an increased risk of developing a host of other psychological and psychiatric problems, including disruptive behaviour problems [92, 93], affective disorders, and anxiety problems [89] compared to either control siblings or population prevalence rates.

Although genetic factors are highly implicated in the etiology of ADHD, environmental factors such as* in utero *chemical toxins, maternal smoking and substance use, low birth weight (a proxy for intrauterine nutrition and growth), and exposure to stress hormones are also operative [7, 94–98]. Psychosocial influences that index the home environment (e.g., quality of parental care, presence of learning materials, and family companionship) have also been shown to be associated with more ADHD-like symptoms in diagnosed children and, to a lesser degree, in their unaffected siblings. The mechanism through which these factors function has yet to be fully mapped, though epigenetic modification of gene expression remains a viable hypothesis [5, 7].

#### 3.2.4. Neuropsychological/Cognitive Impairments of Siblings of Children with ADHD

Compared to the siblings of non-ADHD controls, siblings of children with ADHD who themselves go on to develop ADHD have been shown to demonstrate more neuropsychological difficulties in the areas of attention, executive functioning, and memory [99]. In the latter study, the siblings of children with ADHD who themselves were not diagnosed scored similarly to the siblings of non-ADHD controls. These results suggest that perhaps siblings of children with severe ADHD and associated neuropsychological difficulties are at a particularly heightened risk of later difficulties [88]. However, it is likely that discrete difficulties in certain neurocognitive domains exist for the unaffected siblings of children with ADHD as well [100–102]. For instance, unaffected siblings of children with ADHD are more impaired than controls across executive measures including inhibition, mental shifting, and verbal working memory [100, 102], as well as markers of motivational dysfunction (e.g., reaction time and action monitoring [103–105]). On the other hand, a recent study showed that siblings of children with ADHD were not significantly different than controls on visual-spatial working memory [106]. Thus, similar to ASD, not only is the risk of developing clinical ADHD increased in the siblings of children with the disorder, but also siblings are at risk of showing deficits in a number of endophenotypic neurocognitive domains. Notably, there is evidence that siblings of children with ADHD may “grow out” of early executive deficits [107]. Future prospective studies that track large samples of siblings are needed to determine whether these trends hold for all children or whether there are different pathways to either discrete ADHD diagnosis or the neurocognitive endophenotypes that characterize ADHD.

Finally, it is interesting to note that familial risk of neurocognitive impairment not only is observed in twin studies (see above), but also has been reported in the nontwin siblings of children with ADHD. For instance, there is evidence that children with ADHD and their unaffected siblings demonstrate volume and/or activity reductions in prefrontal, occipital, and parietal regions compared to controls [108]. Further, genetic variability in the dopamine transporter gene (DAT1) has been associated with activation in the striatum and cerebellum in children with ADHD and their unaffected siblings [109]. These results suggest that the genetic liability to ADHD may manifest at the level of neural processing. However, determining when and how these neural and neurocognitive problems manifest at the level of disordered phenotypes is an area that requires further investigation. Indeed, it is plausible that there are multiple pathways to ADHD that rely on unique interactions between genetic and environmental dispositions and their resultant effects on brain development.

### 3.3. Summary and Future Directions for ADHD Sibling Research

Sibling research into ADHD suggests that this relatively common neurodevelopmental disorder is highly heritable and that subclinical or associated impairments may be present in the siblings of children with ADHD, even if a full-blown ADHD diagnosis is not warranted. Compared to research on ASD, the relative paucity of literature using sibling-based designs for ADHD provides important opportunities for future research.

Although early biological and behavioural manifestations of ADHD may have important implications for long-term outcomes [110], there are also a number of individuals with early symptoms of ADHD that resolve with time (i.e., desisters [111]). Given that ADHD symptoms do not in themselves predict developmental outcomes, other factors have the potential to support identification of children at risk of persistent problems [112]. For example, child (e.g., mood dysregulation and language delay) and environmental factors (e.g., parenting and parental mental health) have been associated with persistence of childhood ADHD symptoms [113, 114]. Prospective sibling-based designs allow researchers to explore the trajectory of children at risk as a function of other risk and moderating factors [115]. By comparing high-risk infants who do and do not develop ADHD on differentiating factors, we can further delineate the constitutional and/or environmental moderators that interact to influence the emergence, persistence, and remission of the disorder [116].

## 4. Overlap between ASD and ADHD

Although neurodevelopmental disorders are demarcated as discrete categories in the DSM-5 (in addition to earlier versions), emerging theoretical models and empirical evidence suggest a high degree of overlap between them. A recent review concluded that the cooccurrence of these disorders is high, with studies showing that 20–50% of children with ADHD display variable symptoms of ASD and 30–80% of children with ASD meet criteria for ADHD [117]. Recently, Pourcain and colleagues [118] used a general population sample to study the cooccurring developmental pathways of ADHD and the social communication domain of ASD. They found that over 70% of children in the impaired social communication group were part of the persistently high or moderate hyperactive-inattentive group, and over 80% of children with persistently high hyperactive-inattentive symptoms were included in the impaired social communication group. Further, there were shared prenatal/perinatal risks for social-communication and hyperactive-inattentive trajectories. Further, Cooper et al. [119] investigated 711 children diagnosed with ADHD who did* not* have an ASD diagnosis and found that children with ADHD who had higher levels of autistic symptomatology were more impaired in terms of severity of ADHD and various other domains of psychopathology and cognition. Notably, greater severity in comorbidities was seen after accounting for ADHD symptomatology, suggesting that elevation was related to characteristics of autism rather than being driven by severity of ADHD itself.

With respect to sibling designs specifically, twin studies suggest a moderate degree of overlap in genetic influences on ASD and ADHD traits, whether in the general population or in samples with clinically elevated symptoms [120–122]. In fact, up to 50–70% of the covariance between ASD and ADHD symptoms is explained by additive genetic factors in twin studies. A thorough delineation of the discrete genetic variants associated with both ASD and ADHD is outside the scope of this review, but interested readers may refer to recent empirical and review papers on this topic [123, 124]. Also, Table 1 summarizes several genes that have been implicated in both ASD and ADHD. Although we acknowledge that this list is not exhaustive, it offers a snapshot of the many molecular genetic studies that have been conducted on these disorders, albeit usually in separate investigations. Importantly, DSM-5 has removed ADHD as an exclusionary criterion for an ASD diagnosis, which should usher in a new generation of studies examining both the distinctiveness and the overlap of ASD and ADHD symptoms and whether these are explained by shared genetic or environmental influences.

Currently, sibling studies examining the occurrence of autism symptoms in children with ADHD show that ASD symptoms are more prevalent in children with ADHD than controls. For example, Mulligan et al. [125] examined autism symptoms in a sample consisting of 821 ADHD probands and 1050 siblings, as well as a control group of 149 families. Autism symptoms were higher in children with ADHD compared to siblings and normal controls, and interestingly, ASD symptoms were higher in the affected and unaffected male siblings of children with ADHD compared to controls. This overlap may be best explained at the level of specific cognitive endophenotypes that are shared across these neurodevelopmental disorders.

For instance, Rommelse et al. [126] showed that ASD traits were associated with EF and motor endophenotypes of ADHD, even after correction for ADHD diagnosis. Interestingly, sibling cross-correlations between EF/motor endophenotypes and ASD traits were also significant, suggesting that discrete familial factors which predispose to the neurocognitive deficits in ADHD may also underlie ASD symptomatology. Further, a recent study of 6,595 twins found support for one general genetic factor that accounted for a large proportion of the shared variance (31%) in 53 neurodevelopmental problems characteristic of ASD, ADHD, and other disorders (e.g., motor control, perception, concentration, and tics) [127]. They found particularly high loadings for memory, autistic symptomatology, and ADHD traits. There were also three genetic subfactors specific to impulsivity, learning problems, and tics/autism, as well as three unique environmental factors for autism, hyperactivity and impulsivity, and inattention and learning problems. This study therefore suggests that a common heritable factor may be responsible for multiple neurocognitive problems that typify numerous neurodevelopmental disorders, yet there may also be unique genetic and environmental factors for certain problems. Future studies that elucidate the exact nature of these factors, especially distinct environmental contributors, may provide targets for how to best intervene to promote individual change and foster positive neurocognitive outcomes.

In sum, these varied studies using sibling methodology suggest that not only are ASD and ADHD related through common heritable factors, and perhaps relatively less strongly through environmental conditions, but also there may be specific symptoms that show overlap. Uncovering whether the nontwin siblings of children with particular ASD and ADHD traits also demonstrate increased crossover vulnerability to specific neurocognitive problems using prospective tracking designs is a ripe area for future research.

## 5. Summary of Sibling Research on ASD and ADHD

Taken together, the value of studying the siblings of children with neurodevelopmental conditions such as ASD and ADHD is the ability to (i) determine the relative contribution of genetic and nongenetic factors using twin designs; (ii) quantify the risk of development of these conditions by examining recurrence rates in siblings compared to control siblings or population prevalence rates; (iii) determine whether subclinical impairments in endophenotypes are present, such as social communication, executive functioning, and language, in the siblings of children with neurodevelopmental conditions; (iv) ascertain the risk of other cooccurring psychological/psychiatric conditions; (v) examine the developmental onset of various problems in at-risk siblings by prospectively tracking them and determining when early manifestations of these difficulties first emerge; (vi) identify potential moderating factors that serve to protect children showing early manifestations of difficulties; and (vii) understand the nature of comorbidity and symptom overlap of neurodevelopmental disorders, including how nonspecific neurocognitive traits contribute to various disorders and which neurocognitive traits are explained by common genetic and/or environmental factors. On aggregate, these findings point to a need for appropriate prevention programs in order to reduce the risk of later problems.

## 6. Prevention Programming for At-Risk Children 

Clinically, the ability to identify children at risk of later difficulties (whether disordered in the typical vein or showing relative impairment across the spectrum of symptoms) enables researchers, clinicians, and policymakers to determine who to target for primary prevention given scant societal resources. We know from the above studies that siblings of children with ASD and ADHD represent a subpopulation of children who are vulnerable to suboptimal social, emotional, cognitive, and psychiatric outcomes. We also know that, for ASD in particular, while differentiation of siblings who experience later problems within the first year of life is difficult, there is a constellation of phenotypes that emerge after 12 months that may be key markers of later difficulties. Importantly, there does not appear to be a single atypical behaviour that reliably predicts later neurodevelopmental problems [128]. Rather, there is a heterogeneous set of social-communicative, motor, executive, and sensory behaviours that together predict later diagnosis, all of which need to be considered in prevention efforts.

Prospective studies [55, 60, 61] of children at risk of later problems demonstrate the feasibility of detecting early warning signs through intensive monitoring. Organizations (e.g., American Academy of Pediatrics) have spelled out specific guidelines for the early identification, screening, and diagnosis of neurodevelopmental disabilities, including the suggestion that all children aged 18–24 months be screened for early onset signs [129]. However, the peak for diagnoses of ASD and ADHD is in middle childhood (4–7 years [130, 131]), and the availability of reliable and valid tools for use in children under 3 years remains scarce. Reliance on retrospective parental reports and child histories alone tells a very different story about the development and decline in social communication than prospective surveillance is capable of [62]. These findings speak to the need for active and repeated assessment and discussion about parental concerns [132], as well as the incorporation of developmental observation during regular primary healthcare visits [133]. Although screening tools (e.g., Checklist for Autism in Toddlers [134]) for early signs of atypicality can be used with children under the age of 2 (especially when combined with other surveillance methods), these are rarely used in medical practices [135]. Early screening is particularly indicated for siblings of children with ASD and ADHD, given that they are at higher risk of these and other neurodevelopmental conditions.

Access to early intervention services for those determined to be at risk, by virtue of their family history, genetic disposition, and/or early assessment, is critical to alleviating the burden of neurodevelopmental morbidity on children, families, and society. However, the current state of services for infants and toddlers suspected to have these conditions is limited. Fostering the ability of parents and caregivers to respond sensitively and contingently may be particularly useful for children's emotional, social, and cognitive development [136], an effect that likely holds for both at-risk and typically developing children. For children (including siblings) at risk of neurodevelopmental disorders, interventions that incorporate specific skill building in joint attention, intentional communication with language and gesture, symbolic and reciprocal play, and imitative interactions may be fruitful given that these tend to be areas of difficulty for these children. Importantly, these should be provided in the context of naturalistic environments that involve responsive caregiving, enriched language, and reciprocal interactions that may mitigate the decline in neurodevelopment of at-risk children and foster the functional traits that are critical for psychosocial health and development [132, 137].

Delineating early markers of the emergence and persistence of ASD and ADHD through sibling studies has implications for preventive and early intervention efforts. Infancy and early childhood represent a time of heightened neurobiological sensitivity (i.e., brain plasticity) and thus interventions during this period may have a lasting impact. Additionally, early intervention may serve to derail the onset of maladaptive behavioural patterns associated with symptomatology (e.g., coercive parent-child interactions) and can also support families prior to the formation of negative assumptions and attitudes associated with full symptomatology.

Specific examples of intervention programs for children at risk of neurodevelopmental disorders, defined as being a younger sibling of a child with a neurodevelopmental disorder, are sparse. In fact, we know of none for children at risk of ADHD and could identify only a handful of intervention studies for children at risk of ASD. Three of these were small studies with less than 10 participants: one was a case series of seven siblings of children with ASD [138], while two used multiple-baseline designs of three participants [139, 140]. The best example of parent-mediated interventions for children at familial risk of ASD is captured in a recent study by Green and colleagues [141]. The program was a two-site, two-arm prevention randomized control trial of 54 families (28 intervention; 26 control) of 7–10-month-old infants who had an older sibling with ASD. The intervention was a modification of the Video Interaction for Promoting Positive Parenting program (VIPP [142]), a program that uses video feedback to help parents adjust their parenting styles to the needs of their child to promote positive social and communicative development. The program consisted of six sessions with an additional six booster sessions depending on family needs. Results showed moderate to strong intervention effects on children's attentiveness to their parents, autism-risk behaviours (e.g., behavioural atypicality), attentional disengagement, and parent-reported social-adaptive functioning. Concomitant improvements in parental nondirectiveness (but not sensitivity or mutuality) were also seen. No positive effects for language or responsivity to vowel change were shown. Overall, these results suggest that parent-child interaction-focused interventions may modify the emergence of established behavioural markers of ASD. Moreover, these results are consistent with the general suggestion that early intervention, particularly within the first two years of life, can significantly alter the developmental trajectory of high-risk infants [143].

## 7. Conclusions

Our understanding of neurodevelopmental disorders has increased dramatically over the last four decades, and sibling designs have been instrumental in elucidating the complex and heterogeneous pathways to neurodevelopmental functioning. Twin, recurrence risk, and prospective tracking studies have not only improved our understanding of the etiology of neurodevelopmental conditions but also bolstered our capacity to identify children at risk of particular conditions in order to effectively intervene before the emergence of stable and severe levels of difficulty requires costly remediation. Findings from high-risk sibling studies may thus serve to inform universal screening procedures that could be implemented in primary care settings. These studies could, for example, aid in the development of a risk index that reliably identifies children who could be effectively targeted with early intervention approaches [116]. Once identified, individuals and their families can be referred for treatment services. Child-directed services that target individual profiles may opt to build cognitive skills such as working memory, attention, self-regulation, or social-emotional and communication skills, and there are currently many programs tailored towards such skill building [144–149]. Determining how to successfully adapt these programs to at-risk children who demonstrate subclinical or prodromal symptoms is an area that requires future investigation. Moreover, family-level interventions may support parents to promote cognitive training and/or target parent-child interactional patterns. Such programs are currently being evaluated for children at risk of ASD, and determining whether comparable programs show utility in buffering against ADHD risk is an important question for clinical researchers. As our understanding of the genetic and environmental underpinnings of these disorders continues to expand, movement from targeted to universal screening and intervention may prove feasible, and sibling designs will have undoubtedly served to steer us in the right direction.

## Figures and Tables

**Table 1:** Summary of individual genes that have been implicated in both ASD and ADHD.

Gene	Chromosome	Function	Reference	
			ASD	ADHD
*FMR1 (including premutation) *	Xq28	Involved in making fragile X mental retardation 1 protein (FMRP), an RNA-binding protein involved in gene regulation	[150, 151]	[151–153]

*TSC2 *	16p13	Actin cytoskeleton dynamics; inactivation of GTPase; neuronal migration and cell differentiation	[154–156]	[157, 158]

*NF1 *	17q11	Codes for protein neurofibromin and causally related to neurofibromatosis type 1; associated with inactivation of GTPase; cytoskeleton dynamics	[159, 160]	[161]

*SHANK3 *	22q13	Dendrite morphology regulation; synapse scaffolding and plasticity; binding neuroligins	[162, 163]	[164]

*GRIN2A *	16p13	Codes for the 2A subunit of the NMDA receptor, which is ligand- and voltage-gated and is involved in long-term potentiation and synaptic transmission	[165]	[134, 166]

*GABAR *	15q12	Encodes the GABA receptors and their subunits, thus mediating neurotransmitter inhibitory processes	[167, 168]	[169, 170]^b^

*SLC6A4/5HTT/SERT *	17p11	Encodes the serotonin transporter; mediates reuptake of serotonin from synapses	[171, 172]	[173, 174]

*SLC6A3/DAT/DAT1 *		Encodes a dopamine transporter; regulates extracellular dopamine and reuptake of dopamine from synapses	[175, 176]	[177, 178]

*OXTR *	3p25-26	Encodes the oxytocin receptor and thus mediates the action of oxytocin in various brain regions, particularly those supporting social cognition	[179, 180]	[181]^a^

*AVPR1 *	12q14	Encodes the arginine vasopressin receptor 1a, which mediates cell contraction, proliferation, glycogenolysis, and platelet aggregation	[182, 183]	[184]^c^

*SNAP25 *	20p12-p11.2	Encodes a plasma membrane protein involved in vesicle docking, fusion, and neurotransmitter release	[185, 186]	[178, 187]

*SLC9A9/NHE9 *		Encodes a sodium/proton exchanger; plays a role in cation homeostasis	[188, 189]	[190–192]

*HTR1B *	6q13	Encodes the 5-HT1B receptor, associated with serotonin regulation; depending on location, it is variably involved in modulating serotonin and dopamine in various cortical and noncortical regions	[193]	[194, 195]

*NLGN4 *	Xp22.3	Encodes protein neuroligin 4, involved in cell adhesion, synapse formation, and mediates transsynaptic signalling	[196, 197]	[198, 199]

*DRD4 *	11p15.5	Encodes dopamine receptor D4, a G-protein coupled receptor; upon activation, the receptor inhibits adenylyl cyclase, thus decreasing intracellular cyclic AMP	[200, 201]^d^	[178, 202]

*DRD5 *	4p16.1	Encodes dopamine receptor D5, which is involved in synthesis of cyclic AMP via activation of adenylyl cyclase, and Ca^2+^ and K^+^ conductance	[203]^e^	[178, 204]

*DRD3 *	3q13.3	Encodes dopamine receptor D3; activity is mediated by G-proteins which inhibit adenylyl cyclase; promotes cell proliferation	[205, 206]	[207]

*MAOA *	Xp11.3	Encodes enzymes that deaminate and catabolize neurotransmitters such as dopamine, serotonin, and norepinephrine	[208–210]	[211, 212]

*COMT *	22q11.21	Encodes the catechol-O-methyltransferase enzyme, which catabolizes neurotransmitters such as dopamine and norepinephrine	[213, 214]	[215, 216]

^a^Findings should be considered preliminary.

^b^In this study, *OXTR* was only associated with social cognition in ADHD and not diagnostic status itself.

^c^In this study, *AVPR1A* was associated with executive functioning, not ADHD, although executive functioning is considered a core impairment in ADHD (but also other neurodevelopmental conditions, including autism).

^d^Results may reflect differences in cooccurring symptoms among individuals with ASD as opposed to diagnosis of ASD specifically.

^e^Few studies have shown similar results, so these should be interpreted cautiously.
